# Automatic Classification of Histopathology Images across Multiple Cancers Based on Heterogeneous Transfer Learning

**DOI:** 10.3390/diagnostics13071277

**Published:** 2023-03-28

**Authors:** Kai Sun, Yushi Chen, Bingqian Bai, Yanhua Gao, Jiaying Xiao, Gang Yu

**Affiliations:** 1 Department of Biomedical Engineering, School of Basic Medical Sciences, Central South University, Changsha 410013, China; 2 Department of Pathology, School of Basic Medical Sciences, Central South University, Changsha 410013, China; 3 Department of Ultrasound, Shaanxi Provincial People’s Hospital, Xi’an 710068, China

**Keywords:** artificial intelligence in histopathology, heterogeneous transfer learning, cancer diagnosis, small datasets

## Abstract

Background: Current artificial intelligence (AI) in histopathology typically specializes on a single task, resulting in a heavy workload of collecting and labeling a sufficient number of images for each type of cancer. Heterogeneous transfer learning (HTL) is expected to alleviate the data bottlenecks and establish models with performance comparable to supervised learning (SL). Methods: An accurate source domain model was trained using 28,634 colorectal patches. Additionally, 1000 sentinel lymph node patches and 1008 breast patches were used to train two target domain models. The feature distribution difference between sentinel lymph node metastasis or breast cancer and CRC was reduced by heterogeneous domain adaptation, and the maximum mean difference between subdomains was used for knowledge transfer to achieve accurate classification across multiple cancers. Result: HTL on 1000 sentinel lymph node patches (L-HTL-1000) outperforms SL on 1000 sentinel lymph node patches (L-SL-1-1000) (average area under the curve (AUC) and standard deviation of L-HTL-1000 vs. L-SL-1-1000: 0.949 ± 0.004 vs. 0.931 ± 0.008, *p* value = 0.008). There is no significant difference between L-HTL-1000 and SL on 7104 patches (L-SL-2-7104) (0.949 ± 0.004 vs. 0.948 ± 0.008, *p* value = 0.742). Similar results are observed for breast cancer. B-HTL-1008 vs. B-SL-1-1008: 0.962 ± 0.017 vs. 0.943 ± 0.018, *p* value = 0.008; B-HTL-1008 vs. B-SL-2-5232: 0.962 ± 0.017 vs. 0.951 ± 0.023, *p* value = 0.148. Conclusions: HTL is capable of building accurate AI models for similar cancers using a small amount of data based on a large dataset for a certain type of cancer. HTL holds great promise for accelerating the development of AI in histopathology.

## 1. Introduction

Cancer is a leading cause of death worldwide, with common types including colorectal cancer (CRC), breast cancer, and others. In 2020, the global fatality rates for CRC and breast cancer were 9.4% and 6.9%, respectively [1]. Histopathology is an accurate method for diagnosing cancer [2], but it requires specialized knowledge and clinical experience from pathologists. Unfortunately, there is a shortage of pathologists worldwide, with the number of active pathologists decreasing by 17.53% in the United States from 2007 to 2017 [3]. In low-income countries, such as those in sub-Saharan Africa, there are fewer than one pathologist per 500,000 people [4].

The use of artificial intelligence in histopathology (HAI) has the potential to address the aforementioned limitations, and improve the accuracy and efficiency of diagnosis [5]. For instance, Wang et al. developed an innovative automated AI approach for CRC diagnosis, which achieved a testing accuracy of 98.11% [6]. Kanavati et al. also trained a convolutional neural network based on the EfficientNet-B3 architecture to differentiate between lung carcinoma and non-neoplastic tissues, achieving highly promising results [7]. These achievements have been made possible by leveraging deep learning methods, which require massive amounts of data collection and annotation. For instance, ref. [6] gathered 14,680 whole slide images (WSIs) from 9631 subjects and labeled 170,099 patches. Similarly, ref. [7] utilized a dataset of 3704 WSIs acquired from Kyushu Medical Centre for training and validation purposes. Furthermore, data preparation must be repeated for each cancer, resulting in an extremely heavy workload and becoming a bottleneck for HAI.

Recently, significant progress has been made in reducing the number of annotations [8], including semi-supervised [9,10,11] and unsupervised learning [12,13,14]. However, despite these advancements, large amounts of unlabeled images are still needed [15]. The use of generative adversarial networks for data generation has shown promise in decreasing the amount of annotation and data collection required [16,17,18]. However, the generated data is often limited by the existing data distribution [19], which can lead to the generation of incorrect or misleading data that can negatively impact the training of the model [19]. Additionally, most studies focused on a single type of cancer [5,20], necessitating repetitive data preparation for each new type of cancer.

In fact, some cancer cells from different types of cancer share similar characteristics and features, such as large nuclei and strong adhesion among cells [21], indicating the potential for building AI models across multiple cancer types. Heterogeneous transfer learning (HTL) [22] is a method that transfers these similar features between different distributed datasets and has been widely applied in natural images and some medical images, such as CT images [23] and MR images [24]. However, its effectiveness in histopathology images has not yet been proven.

We discuss here three cancers including CRC, breast cancer, and sentinel lymph node metastasis, all originating from glandular epithelium and falling under the category of adenocarcinoma. These cancers display similar tissue morphology and structure, such as the shape of cancer nests, morphology of single cancer cells, and overlapping molecular phenotypes. Furthermore, the interstitium of these carcinomas also share similarities [21].

An HTL framework is proposed in this study. The framework extracts general features of cancer cells from NCT-CRC-HE-100K, a large CRC dataset [25], and transfers them to the classification task of sentinel lymph node metastasis and breast cancer. The framework only uses a small number of labeled images [26,27] for training across multiple cancers and demonstrates that a robust model can be obtained by incorporating features from CRC. The main contributions of this study can be summarized as follows:

(1) We demonstrate that features extracted from CRC can aid in the learning of lymph node metastasis and breast cancer, potentially reducing the amount of data needed for these cancer types;
(2) The presented HTL method demonstrates generalizability across different types of cancers and has the potential to accelerate the development of HAI.

## 2. Methods

### 2.1. Datasets

We utilized three different datasets comprising of three types of cancers, namely NCT-CRC-HE-100K [25], Camelyon16 [26], and BreaKHis [27]. NCT-CRC-HE-100K is a large dataset containing 100,000 non-overlapping patches of size 224 × 224, derived from 86 Hematoxylin-eosin (H&E) stained WSIs of CRC. Out of the 100,000 patches, 14,317 patches are malignant and 85,683 patches are benign. 

The Camelyon16 dataset is composed of 399 H&E stained WSIs of sentinel lymph nodes, divided into 270 training and 129 testing WSIs. The training set comprises of 160 benign WSIs and 110 WSIs containing malignant tumor tissue, while the test set contains 80 benign WSIs and 49 WSIs containing malignant tumor tissue. It should be noted that sentinel lymph node metastasis is a consequence of breast cancer cells spreading to lymph nodes.

**Figure 1:**
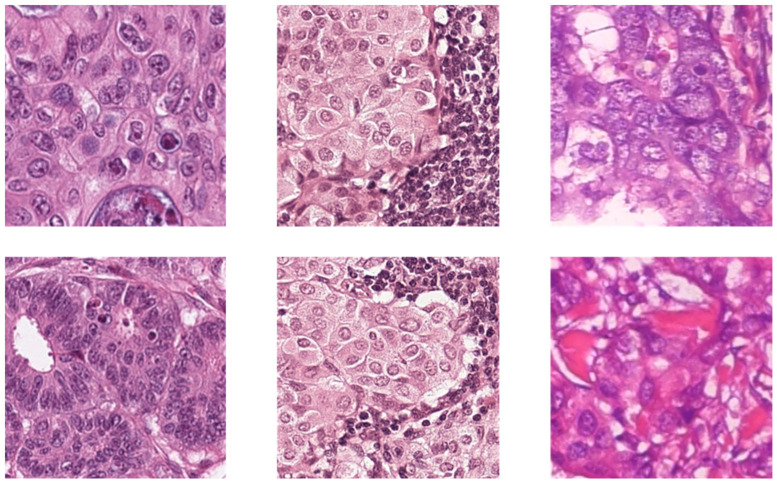
The sample images of three datasets. The columns from left to right are CRC, sentinel lymph node metastasis and breast cancer.

The BreaKHis dataset consists of 2013 images of size 700 × 460, acquired at 20× magnification from 82 subjects’ H&E stained WSIs. Out of the 2013 images, 623 are benign from 24 subjects, and the other 1390 images are malignant from 58 subjects. More detailed information and sample images of the three datasets can be found in Table 1 and Figure 1, respectively.

### 2.2. Data Preprocess Pipeline

We utilized all 14,317 malignant patches and randomly selected 14,317 benign patches from the NCT-CRC-HE-100K dataset to construct a balanced dataset (Dataset-CRC). In this study, Dataset-CRC serves as the source domain dataset, and all of its samples were used as the training set for training the CRC model of source domain.

The Camelyon16 dataset has fixed WSIs for training and testing. In the benign WSIs, all tissue regions are cut into non-overlapping 300 ∗ 300 patches, while in malignant WSIs, only malignant tumor tissue regions are used to extract the patches. To avoid extracting excessive redundant patches and to balance the number of malignant and benign patches, we randomly select 40 patches from each malignant WSI and 28 patches from each benign WSI in the training set. Furthermore, the patches are divided into a training set and a validation set based on an 8:2 ratio of the WSIs. Moreover, we used all 54,105 malignant patches and 54,014 randomly selected benign patches from the test set to evaluate the performance of the model. These patches were used to create the Dataset-SLN.

Non-overlapping patches are extracted from 2013 images of 82 patients in the BreaKHis dataset, resulting in 3738 benign patches and 8340 malignant patches. From the 8340 malignant patches, 3738 patches are randomly selected and combined with all 3738 benign patches to form Dataset-BRE. These patches are then divided into training, validation, and test set at a ratio of 7:1:2, ensuring that patches from the same patient do not appear in multiple sets. The preprocessed three datasets are shown in Table 2.

### 2.3. HTL Framework

The HTL framework proposed in this study comprises of two modules, namely the source domain model and the target domain model, both of which utilize Resnet50 [28]. Each module includes a feature extractor and two fully connected layers (FCs). The feature extractor is composed of several bottleneck residual blocks that output a 2048-dimensional feature vector. The FCs are used to convert the feature vector into categories, starting with 2048 dimensions and reducing it to 256 dimensions, and finally classifying it into two categories—benign or malignant cancer.

**Figure 2:**
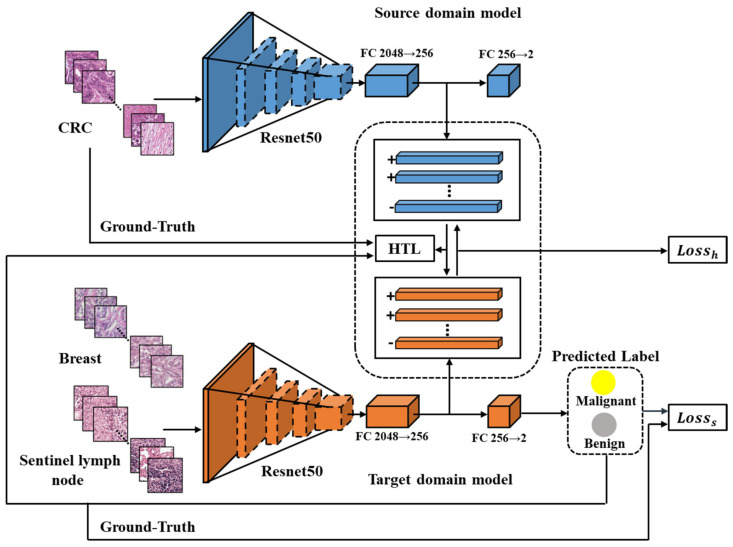
The flowchart of the proposed HTL framework. Resnet50 is depicted in blue as the source domain model and in orange as the target domain model. The CRC features are extracted from the source domain model, while the features of breast or sentinel lymph node are extracted from the target domain model for computing the HTL loss during domain adaptation. The ‘+’ and ‘−’ symbols indicate the feature vectors from malignant and benign samples, respectively.

As illustrated in Figure 2, the source domain model has been trained end-to-end using Dataset-CRC to extract general features of CRC. A target domain model is developed for each of the other cancers. The input images of both models undergo conventional image augmentation techniques such as resizing, random horizontal flipping, random cropping, and normalization [29]. Patches of CRC are fed into the trained source domain model to obtain 256-dimensional features, while patches of breast or sentinel lymph node are input into the target domain model to obtain predicted labels and 256-dimensional feature vectors. The HTL loss, computed using an improved Maximum Mean Discrepancy (MMD) method [22], aligns the features across cancers based on the 256-dimensional vectors from CRC and breast or sentinel lymph node. Moreover, the supervised loss guides the output of the target domain model to be consistent with the labels of breast or sentinel lymph node.

#### Cross-Cancer Domain Adaptation Using HTL Operation

The traditional MMD performs global alignment between the source and target domains without considering the distributions of different categories within each domain. This may not effectively transfer the differences between the benign (normal tissues) and malignant (cancerous tissues) categories [30,31]. Since the features of benign and malignant categories are distinctly different, global alignment may cause confusion between them, resulting in incorrect HTL operation.

**Figure 3:**
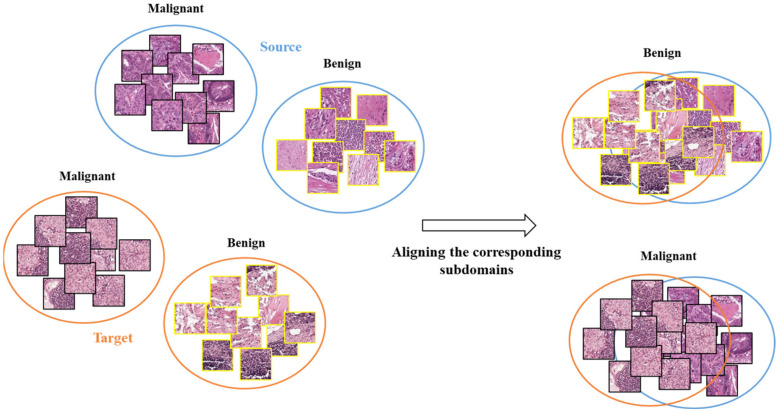
The schematic diagram for illustrating subdomain adaptation.

Our proposed HTL operation across cancers involves aligning the distributions of subdomains (i.e., categories) to perform effective feature transfer. Unlike traditional MMD, which performs global alignment without considering differences between categories in two domains, our HTL operation reduces the feature distribution differences between CRC and sentinel lymph node metastasis or breast cancer, as depicted in Figure 3.

The HTL loss is calculated using the improved MMD, which is defined as:

(1) $${\mathrm{Loss}}_{h}=\frac{1}{C}\sum_{c=1}^{C}{\left\|\sum_{i=1}^{n}w_{i}^{t}\phi \left(T_{i}\right)-\sum_{j=1}^{m}w_{j}^{s}\phi \left(S_{j}\right)\right\|}_{H}^{2}$$

where c represents the benign or malignant category, t and s indicate the source domain and target domain respectively, n and m are the numbers of samples in a batch of source domain and target domain, H represents Hilbert space, ϕ is a mapping function that transforms the features of Euler space to the Hilbert space, $S_{j}$ and $T_{j}$ are 256-dimensional feature vectors that represents CRC and the target domain (either sentinel lymph node metastasis or breast cancer), respectively, $w_{j}^{s}$ and $w_{i}^{t}$ are the weights of the category of $S_{j}$ and $T_{j}$, that are calculated as follows:

(2) $$w_{i}^{c}=\frac{y_{i}}{\sum_{i=1}^{n}y_{i}}$$

For the source domain, the one-hot vector $y_{i}$ is derived from the actual label of CRC, which takes a value of 0 (benign) or 1 (malignant). For target domain, $y_{i}$ refers to the predicted class probability for sentinel lymph node metastasis or breast cancer, generated by the target domain model.

The SL loss is used for supervised learning of sentinel lymph node metastasis or breast cancer. It is obtained by calculating the cross-entropy loss between the predicted probability distribution of classes and the ground-truth labels of sentinel lymph node metastasis or breast cancer, as defined in Equation (3).

(3) $${\mathrm{Loss}}_{s}=-\frac{1}{n}\sum_{i=1}^{n}\left[y_{i}\log\left(p_{i}\right)+(1-y_{i})\log(1-p_{i})\right]$$

where n is the number of samples in a batch, $y_{i}$ and $p_{i}$ denote the actual label and the predicted probability, respectively.

The total loss function is the weighted sum of SL loss and HTL loss.

(4) $${\mathrm{Loss}}_{t}={\mathrm{Loss}}_{s}+\alpha [g\left(\mathrm{epoch}\right){\mathrm{Loss}}_{h}]$$

where ${\mathrm{Loss}}_{t}$, ${\mathrm{Loss}}_{s}$ and ${\mathrm{Loss}}_{h}$ represent the total loss, SL loss and HTL loss, respectively, α is constant coefficient, and $g\left(\mathrm{epoch}\right)$ is a monotonically increasing function of the number of epochs, defined by Formula (5).

(5) $$g\left(\mathrm{epoch}\right)=\frac{2}{1+e^{-10\frac{\mathrm{epoch}}{\mathrm{nepoch}}}}-1$$

where e is the Euler number and nepoch represents the total epoch.

### 2.4. Experiment Setting

To demonstrate that HTL can reduce the amount of labeled data required, HTL is needed to be compared with massively labeled supervised learning (SL) as well as SL with insufficient labeled data. Moreover, HTL models trained with a small number of labeled data should perform comparably to massively labeled training models and significantly outperform models trained with insufficient labeled data.

Therefore, we trained three different versions of models for each cancer: one HTL version and two SL versions (SL-1 and SL-2). SL-1 is trained on insufficient labeled data, while SL-2 is trained on sufficient labeled data. The code is implemented in PyTorch (version 1.8) [32] and runs on a graphics processing unit (GPU) of Tesla V100 32 GB (NVIDIA company, Santa Clara, CA, USA). We compared the performance of Resnet18, Resnet50, and Resnet101 and found that Resnet50, initialized on ImageNet [33], achieved the best performance.

#### 2.4.1. Sentinel Lymph Node Metastasis Models

The models for sentinel lymph node metastasis include L-HTL-1000, L-SL-1-1000, and L-SL-2-7104. L-HTL-1000 and L-SL-1-1000 use the same training and validation set, which consists of approximately 13% of all training and validation patches. L-SL-2-7104 is trained and validated using 7104 and 1776 patches, respectively. The test set for all three models comprises 108,119 patches. Table 3 shows the number of patches used for training, validation, and testing for each model.

#### 2.4.2. Breast Cancer Models

The breast cancer models consist of three models: B-HTL-1008, B-SL-1-1008, and B-SL-2-5232. B-HTL-1008 is trained and validated with 1008 and 114 patches, respectively, which account for approximately 19% of all training and validation patches. B-SL-1-1008 uses exactly the same data as B-HTL-1008 for training and validating. Additionally, B-SL-2-5232 is trained and validated with all patches in the training and validation set. The test set comprises 1496 patches and is used to evaluate the performance of all three models.

The dataset was randomly split, and each model was trained eight times for cross-validation. The hyperparameter selection process for these models was the same, and various hyperparameters were tested, including learning rate (0.05, 0.01, 0.015), batch size (16, 32, 64), and others, until the model’s performance was optimal. The hyperparameter settings for SL-1 and SL-2 were consistent with the HTL version. Additionally, the SL-2 version only increased the number of samples in the training set for two datasets compared to the SL-1 version, while the others remained the same. Detailed hyperparameters are listed in Table 4.

## 3. Results

### 3.1. Classification of CRC, Breast and Sentinel Lymph Node Metastasis by Source Domain Model

In order to compare the difference between CRC, sentinel lymph node metastasis and breast cancer, we tested the source domain model on them, where the CRC-VAL-HE-7K is provided alongside NCT-CRC-HE-100K for CRC testing purposes [25]. The results are shown in Table 5. The AUC, accuracy, sensitivity and specificity are 0.986, 0.948, 0.951 and 0.944, respectively, which show that the source domain model can accurately identify CRC. In contrast, this model struggled to effectively identify breast cancer and sentinel lymph node metastasis.

These results indicate that despite all three cancers being adenocarcinomas, their image features differ. While the source model trained on CRC can achieve high accuracy for CRC, it falls short for breast cancer and sentinel lymph node metastasis. Moreover, the significant difference in AUC for breast cancer and sentinel lymph node metastasis suggests that although lymph node metastasis originates from breast cancer, there may be morphological changes between the metastatic and primary cancer.

The results across multiple cancers are also provided in Section 3.2 and Section 3.3, where we compare the performance of the three models (SL-1, HTL, and SL-2) for each cancer. The two SL versions describe the model differences trained on a small dataset and large dataset, respectively, while the HTL version shows how CRC image features can improve performance on small datasets through domain adaptation. We report the area under the curve (AUC) to demonstrate the comprehensive performance of all models, as well as accuracy, sensitivity, specificity, F1 score and precision. The eight-fold cross-validation for three models is performed for statistical comparisons. All presented results are based on patch-level analysis.

### 3.2. Classification of Sentinel Lymph Node Metastasis

**Figure 4:**
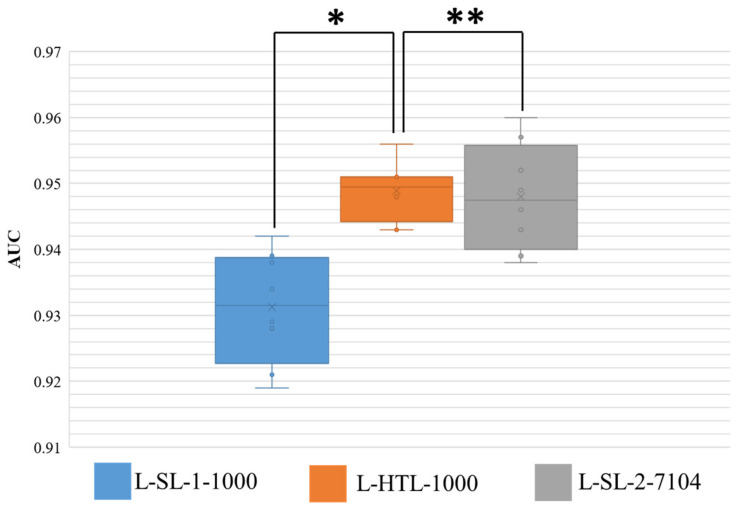
This figure displays the distribution of area under the curve (AUC) for the three models on Dataset-SLN. The ** symbol indicates no significant statistical difference, while * indicates that there is a statistical difference. The boxes represent the upper and lower quartile values, and the whiskers indicate the minimum and maximum values. The horizontal bar within the box denotes the median, while the cross denotes the mean. The circles represent data points, while the scatter dots indicate outliers.

The results of eight-fold cross-validation on Dataset-SLN are presented in Figure 4, where the area under the curve (AUC) is shown. The Wilcoxon-signed rank test is performed on the results, and two-sided *p* values are reported. The HTL version trained on 1000 sentinel lymph node patches (L-HTL-1000) outperformed the SL-1 version trained on the same data (L-SL-1-1000) with an average AUC and standard deviation of 0.949 ± 0.004 vs. 0.931 ± 0.008, respectively (*p* value = 0.008). Moreover, there was no significant difference between the performance of L-HTL-1000 and L-SL-2-7104 (AUC: 0.949 ± 0.004 vs. 0.948 ± 0.008, *p* value = 0.742). These results further confirm the excellent performance of HTL on small datasets.

**Figure 5:**
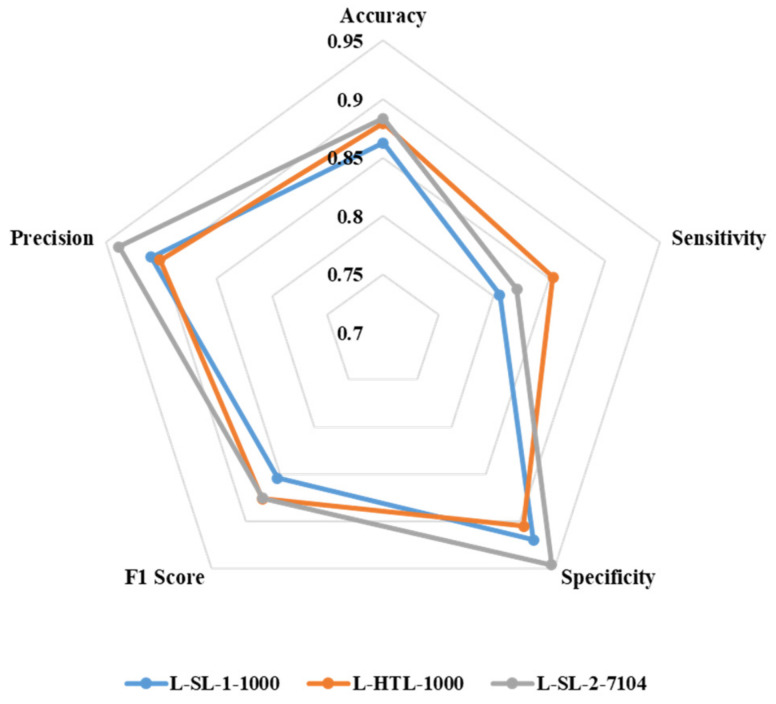
The radar chart is used to illustrate the accuracy, sensitivity, specificity, F1 score, and precision of L-SL-1-1000, L-HTL-1000, and L-SL-2-7104.

The accuracy, sensitivity, specificity, F1 score, and precision of L-SL-1-1000, L-HTL-1000, and L-SL-2-7104 are presented in Figure 5. L-HTL-1000 shows a higher accuracy (0.879) compared to L-SL-1-1000 (0.862) and is close to L-SL-2-7104 (0.883). Additionally, L-HTL-1000 exhibits the best sensitivity of 0.854, which is 0.049 higher than L-SL-1-1000 (0.805) and 0.033 higher than L-SL-2-7104 (0.821). However, L-HTL-1000 has lower specificity (0.905) than L-SL-1-1000 (0.919) and L-SL-2-7104 (0.946). The F1 score of L-SL-1-1000, L-HTL-1000, and L-SL-2-7104 is 0.854, 0.876, and 0.875, respectively, and the precision is 0.909, 0.901, and 0.938, respectively. These results are obtained from the average of eight-fold cross-validation and based on the Youden index [34] as the cut-off.

### 3.3. Classification of Breast Cancer

**Figure 6:**
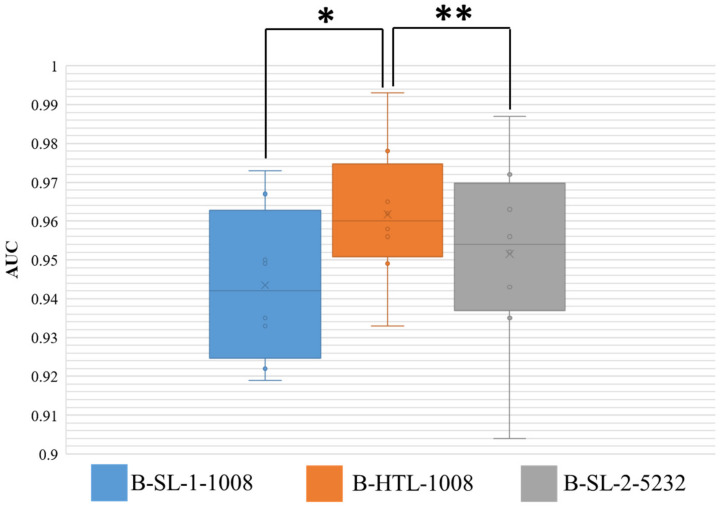
AUC distribution of three models on Dataset-BRE. The ** denotes no statistically significant difference, while * denotes a statistically significant difference. The boxes indicate the upper and lower quartile values, and the whiskers indicate the minimum and maximum values. The horizontal bar in the box indicates the median, while the cross indicates the mean AUC. The circles represent data points, and the scatter dots indicate outliers.

The AUC results for Dataset-BRE are presented in Figure 6, and the Wilcoxon-signed rank test was conducted on the results of eight-fold cross-validation with two-sided *p* values reported. The HTL on 1008 breast patches (B-HTL-1008) demonstrated superiority over supervised learning on the same dataset (B-SL-1-1008), with an average AUC and standard deviation of 0.962 ± 0.017 vs. 0.943 ± 0.018, respectively, and a *p* value of 0.008. Furthermore, there was no significant difference between the HTL on 1008 patches (B-HTL-1008) and SL on 5232 patches (B-SL-2-5232), with AUCs of 0.962 ± 0.017 and 0.951 ± 0.023, respectively, and a *p* value of 0.148. These results indicate that HTL performs better than SL when the amount of data is small and can achieve comparable performance to that of large datasets.

**Figure 7:**
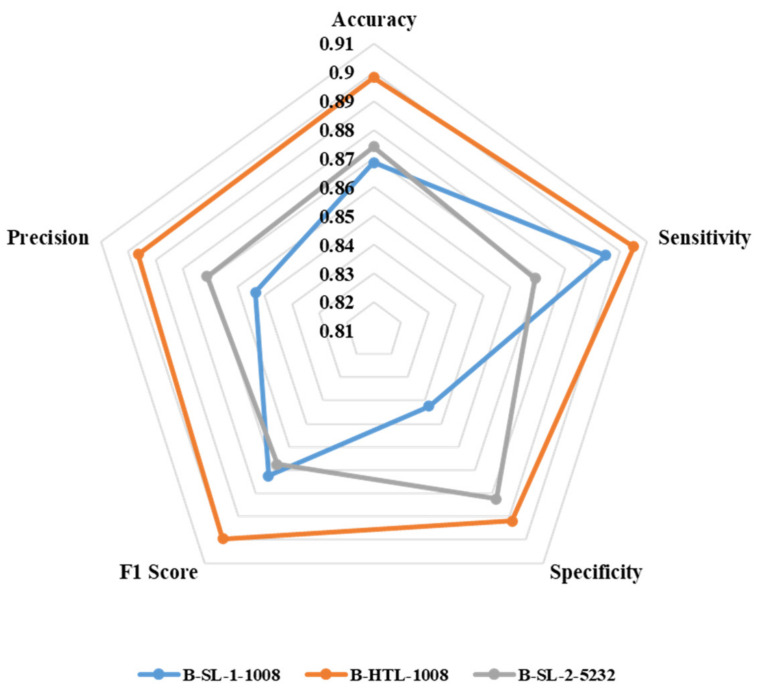
The radar chart for illustrating the accuracy, sensitivity, specificity, F1 score and precision of B-SL-1-1008, B-HTL-1008 and B-SL-2-5232.

Figure 7 displays the average values of eight-fold cross-validation for accuracy, sensitivity, specificity, F1 score, and precision. B-HTL-1008 exhibits superior accuracy (0.898) compared to B-SL-1-1008 (0.869) and B-SL-2-5232 (0.874). Additionally, B-HTL-1008 has the highest sensitivity and specificity (0.905 and 0.892), which surpasses B-SL-1-1008 by 0.010 and 0.049 (with values of 0.895 and 0.843, respectively), and B-SL-2-5232 by 0.036 and 0.010 (with values of 0.869 and 0.882, respectively). The F1 scores of B-SL-1-1008, B-HTL-1008, and B-SL-2-5232 are 0.872, 0.900, and 0.867, while their corresponding precision values are 0.853, 0.896, and 0.871. It is worth noting that the results are optimized through the Youden index [34].

## 4. Discussion

Histopathology is a critical component of clinical diagnosis, and while HAI holds promise as an effective tool for improving diagnostic accuracy and reducing misdiagnosis resulting from heavy workloads or limited pathologists, the cost of data preparation for model establishment has become a bottleneck in HAI development.

While techniques such as semi-supervised and unsupervised learning can help decrease the cost of data annotation, the collection of massive amounts of unlabeled data remains a necessity. Furthermore, obtaining enough samples of each type of cancer can be challenging or even impossible in clinical practice due to a shortage of disease-specific samples. 

The histopathological diagnosis of cancer relies on examining the morphology and tissue structure of cancer cells [21]. We postulate that deep learning can detect similarities in image features across different cancers. Specifically, a feature extractor from a highly accurate source model built on a large cancer dataset may offer general image features for cancers, which could reduce the required amount of data and facilitate model construction for other types of cancers. 

Given the hypothesis that HTL could enhance AI model training for similar cancers, we chose to examine CRC, breast cancer, and lymph node metastasis. These cancers all originate from epithelial tissue and fall under adenocarcinoma, demonstrating comparable tissue morphology and structure such as cancer nest shape, individual cancer cell morphology, and overlapping molecular phenotypes. 

We first built a model of the source domain based on a large CRC dataset. Although breast cancer and sentinel lymph node metastasis, like CRC, are both adenocarcinomas, the CRC model cannot effectively recognize the former two types of cancer, indicating that the source domain model considers the image features of breast cancer and sentinel lymph node metastasis to be different from those of CRC. Moreover, the CRC model shows a significant difference in AUC for breast cancer and sentinel lymph node metastasis, which suggests that although lymph node metastasis originates from breast cancer, there may be morphological changes between the metastatic and primary cancer. 

When using a certain amount of breast cancer and sentinel lymph node metastasis images and combining them with the CRC model in heterogeneous transfer learning, precise classification results for the first two types of cancer can be achieved. However, without using the CRC model, the performance of the models trained on these images would significantly decrease. These experiments may demonstrate that the colon cancer model can provide some common image features of adenocarcinomas, while the images of other adenocarcinomas provide unique image features for each specific type of adenocarcinoma. Heterogeneous transfer learning can integrate both types of features to obtain accurate recognition models for other adenocarcinomas, similar to the results of massive labeled SL.

Our work demonstrates that when there is an accurately trained HAI based on a large dataset, it is not necessary to collect and label a large amount of data for other similar cancers. Therefore, HTL can reduce the data and labeling costs of these cancers, especially for some cancers that are difficult to obtain data for. In clinical practice, it is often observed that a large amount of data has been collected for one type of cancer, but not enough data has been collected for similar types of cancer, Therefore, HTL has broad application prospects. 

We have demonstrated, for the first time, that the presented HTL method has the potential to quickly develop HAI models for similar cancers by reducing the amount of required data. However, a main limitation of this study is the limited number of cancer types and validation data. In future studies, we aim to investigate the applicability of the HTL method to other cancers to further validate our findings. If HTL can be widely applied to learning across cancers, it may overcome the data bottleneck and accelerate the deployment of HAI across diseases. 

## 5. Conclusions

We proposed a novel HTL approach for HAI across various cancers. We conducted experiments on publicly available datasets for sentinel lymph node metastasis and breast cancer and demonstrated that our proposed method can create high-accuracy models using limited datasets by transferring features across different types of cancer. Our findings verify the ability of HTL to reduce data volume in the target domain, indicating its potential for deployment in HAI applications. 

## Figures and Tables

**Table 1:** Datasets for three types of cancer.

Dataset Name		Slides/Patients			Images/Patches		
		Malignant	Benign	Total	Malignant	Benign	Total
NCT-CRC-HE-100K		NA	NA	86	14,317	85,683	100,000
Camelyon16	Training set	110	160	270	NA	NA	NA
	Test set	49	80	129			
BreaKHis		58	24	82	1390	623	2013

**Table 2:** The preprocessed datasets.

Domain	Dataset Name	Dataset Usage	Type	Slides/Patients	Patches
Source domain	Dataset-CRC	Training set	Malignant	NA	14,317
			Benign	NA	14,317
			Total	86	28,634
Target domain	Dataset-SLN	Training set	Malignant	88	3520 *
			Benign	128	3584 *
			Total	216	7104
		Validation set	Malignant	22	880 *
			Benign	32	896 *
			Total	54	1776
		Test set	Malignant	49	54,105
			Benign	80	54,014
			Total	129	108,119
	Dataset-BRE	Training set	Malignant	42	2616
			Benign	18	2616
			Total	60	5232
		Validation set	Malignant	5	374
			Benign	2	374
			Total	7	748
		Test set	Malignant	11	748
			Benign	4	748
			Total	15	1496

* Because the number of pixels of various WSIs can vary significantly (especially since the area of malignant tumor tissue in WSI is quite different), the number of patches is estimated by the randomly selected number of patches from one WSI.

**Table 3:** Patches in the training, validation and test set for three models on two datasets.

Datasets			HTL	SL-1	SL-2
Dataset-SLN	Training	Malignant	500	500	3520
		Benign	500	500	3584
		Total	1000	1000	7104
	Validation	Malignant	100	100	880
		Benign	100	100	896
		Total	200	200	1776
	Test	Malignant	54,105	54,105	54,105
		Benign	54,014	54,014	54,014
		Total	108,119	108,119	108,119
Dataset-BRE	Training	Malignant	504	504	2616
		Benign	504	504	2616
		Total	1008	1008	5232
	Validation	Malignant	57	57	374
		Benign	57	57	374
		Total	114	114	748
	Test	Malignant	748	748	748
		Benign	748	748	748
		Total	1496	1496	1496

**Table 4:** Hyperparameters used in our model.

Hyperparameters	Value
Optimizer	SGD
Epochs	200
Momentum	0.9
L2 weight decay	0.0005
Learning rate	0.01
Batch size	32

**Table 5:** The results of source domain for identifying CRC, breast cancer and sentinel lymph node metastasis.

Dataset	AUC	Accuracy	Sensitivity	Specificity
CRC-VAL-HE-7K	0.986	0.948	0.951	0.944
Dataset-SLN (Test set)	0.692	0.540	0.009	0.986
Dataset-BRE (Test set)	0.307	0.304	0.004	0.991

## Data Availability

The Camelyon16 dataset can be find in: https://camelyon16.grand-challenge.org/Home/, the NCT-CRC-HE-100K and CRC-VAL-HE-7K dataset can be find in: https://www.zenodo.org/record/1214456#.ZCFCDcpBxD8 and the BreakHis dataset can be find in: https://web.inf.ufpr.br/vri/databases/breast-cancer-histopathological-database-breakhis/.
